# On the MAC/Network/Energy Performance Evaluation of Wireless Sensor Networks: Contrasting MPH, AODV, DSR and ZTR Routing Protocols

**DOI:** 10.3390/s141222811

**Published:** 2014-12-02

**Authors:** Carolina Del-Valle-Soto, Carlos Mex-Perera, Aldo Orozco-Lugo, Mauricio Lara, Giselle M. Galván-Tejada, Oscar Olmedo

**Affiliations:** 1 Department of Electrical and Computer Engineering, Tecnologico de Monterrey, Ave. Eugenio Garza Sada #2501 Sur, Monterrey NL, 64849, Mexico; E-Mail: carlosmex@itesm.mx; 2 Center for Research and Advanced Studies of IPN, Communications Section, Av. IPN No.2508, Colonia San Pedro Zacatenco, CP 07360, Mexico D.F., Mexico; E-Mails: aorozco@cinvestav.mx (A.O.-L.); mlara@cinvestav.mx (M.L.); ggalvan@cinvestav.mx (G.M.G.-T.); oolmedo@cinvestav.mx (O.O.)

**Keywords:** wireless sensor networks (WSNs), performance metrics, energy consumption

## Abstract

Wireless Sensor Networks deliver valuable information for long periods, then it is desirable to have optimum performance, reduced delays, low overhead, and reliable delivery of information. In this work, proposed metrics that influence energy consumption are used for a performance comparison among our proposed routing protocol, called Multi-Parent Hierarchical (MPH), the well-known protocols for sensor networks, Ad hoc On-Demand Distance Vector (AODV), Dynamic Source Routing (DSR), and Zigbee Tree Routing (ZTR), all of them working with the IEEE 802.15.4 MAC layer. Results show how some communication metrics affect performance, throughput, reliability and energy consumption. It can be concluded that MPH is an efficient protocol since it reaches the best performance against the other three protocols under evaluation, such as 19.3% reduction of packet retransmissions, 26.9% decrease of overhead, and 41.2% improvement on the capacity of the protocol for recovering the topology from failures with respect to AODV protocol. We implemented and tested MPH in a real network of 99 nodes during ten days and analyzed parameters as number of hops, connectivity and delay, in order to validate our simulator and obtain reliable results. Moreover, an energy model of CC2530 chip is proposed and used for simulations of the four aforementioned protocols, showing that MPH has 15.9% reduction of energy consumption with respect to AODV, 13.7% versus DSR, and 5% against ZTR.

## 1. Introduction

Wireless sensor networks (WSNs) consist of a number of spatially-distributed autonomous devices using sensors to monitor physical or environmental conditions. They are multi-functional, low-cost and low-power networks and rely on communications among nodes or from sensor nodes to one or more sink nodes. Sink nodes, sometimes called coordinator nodes or root nodes, may be more robust and have larger processing capacity than the other nodes. Sensor networks can be widely used in various environments, sometimes hostile. Some of the many applications of WSNs are: the medical field, agriculture, monitoring and detection, automation and data mining [1].

The most notable issues regarding WSNs are the difficulty in transmitting information in a wireless environment, as well as the energy costs implied. The signal fades, obstacles, channel occupancy and interference with other devices motivate the use of powerful mechanisms to send and receive packets successfully. Besides, there are other particular problems in WSNs related to the memory and processing. These networks have limited resources because of the cost and size of the devices. The sensors are small in order to be adaptable to all kinds of environments and able to be installed in various conditions, locations and infrastructures. This also causes the batteries to be small and short-lived; thus, herein is the need to save energy in all processes of the network.

Due to the wireless conditions, topology dynamics must be taken into account. The time it takes for the network to reconfigure itself will be directly affected by the transmitted and dropped packets. Likewise, the connection and disconnection of nodes alters the packet loss and the rearrangement of the network topology. Here, the routing protocol must provide some rules aimed at reorganizing the links and the topology, and it might also need to establish new routes as default routes. However, these failures have to be only temporary and the protocol must be able to return the network to its stable state in a very short time [2].

Regarding the energy problem, there are specific factors that directly affect node battery consumption. The term unbalanced energy depletion, shown in [3], describes a situation where the nodes that are closer to the coordinator node carry more traffic, and so, they consume more energy than those nodes further away from the root node. This imbalance causes the overall energy consumption to be distributed non-uniformly in the network, making some nodes run out of energy faster than others.

A fundamental aspect of node energy consumption is the coverage radio, because it influences the amount of neighbors that a node has and its capacity to forward packets. This coverage should, ideally, reach several strategic points in the network to send information, but it should not affect overhead and energy costs. Chen *et al.* apply probabilistic methods to find the ideal coverage of distributed nodes along the network. They evaluate the network energy performance based on the lifetime of the sensors and the balance of the energy among them, achieving strategic point location of sensors [4]. Likewise, the work in [5] discusses a method to find the ideal radius considering the average number of neighbors that a node must have in order to forward traffic to the coordinator node without creating overhead and higher energy consumption in the network.

Another issue that influences battery consumption is the hop count from source to destination. If the routing protocol finds the shortest routes, then the energy consumption to carry a packet from a source to its destination will be reduced. This is where protocols, such as AODV [6] and DSR [7] optimize energy depletion by choosing the best routes, i.e., routes with a lesser hop count. Some authors describe these matters in [8–10].

Factors, such as the number of retransmissions and retries when the MAC layer is listening to the channel, delays and general overhead also have an indirect influence over the total energy consumption, and they give an indicator of the network performance. In our work, we discuss these factors to show the efficiency of each of the protocols presented here. The type of transmission, reception, CSMA/CA (carrier sense multiple access with collision avoidance) algorithm, switching and listening in the MAC layer affects the energy problem, and they are well depicted and described in [11]. The problem of the local energy consumed by each node is treated in [12]. This model takes into account sensing, receiving and transmitting energies, and the network is partitioned into rings to analyze the behavior of the nodes according to the proximity to the sink or collector node.

In this paper, the authors propose an energy model and use it to compare the performance of AODV, DSR and ZTR and the proposed multi-parent hierarchical (MPH) protocol [13] in the context of a single-sink scenario consisting of static nodes that send and receive information to and from a coordinator node. We assume that all nodes, except for the coordinator, have limited energy, memory and processing resources. A hierarchical logic topology fits well in this scenario: packets are transmitted from source nodes to the coordinator node by establishing routes where each next hop belongs to a nearest neighbor node (or nodes). The main objective is to analyze the principal MAC and network layer metrics that directly affect energy consumption for AODV, DSR, ZTR and MPH. To this end, the proposed energy model provides a way to compute the total consumed energy in the network, globally and locally. We will see that our MPH protocol, purposed to be built for these conditions, is more efficient and has a lower cost than the other protocols under evaluation.

We now describe the organization of the rest of the paper. In Section 2, we describe related work. Section 3 reviews the efficient multi-parent hierarchical (MPH) routing protocol and shows a physical network implementation under the MPH protocol. Some parameters are considered in order to compare the results of the real implementation with those of our simulator. Section 4 discusses the results of the proposed metrics classified into: the MAC layer (retransmissions and CSMA/CA retries), network layer (overhead, discovered and valid routes) and global energy metric; the evaluated protocols are AODV, DSR, ZTR and MPH. This section also details the proposed energy model used to calculate the global and local energy consumption. In Section 5 the protocols are evaluated under different topology arrangements. Finally, conclusions are given in Section 6.

## 2. Related Work

Currently, communications make individuals take part of a whole world. The Internet of Things (IoT) is a concept that includes people, communications protocols and devices for the purpose of easy interaction and countless applications. Due to the complexity of this type of scenario, WSNs are suitable for deployment and implementation. This is where control parameters, such as the management of resources and energy, traffic congestion, automation, device independence and quality of service, become relevant [14]. WSNs have a dynamically changing nature, because nodes operate in a wireless medium, and connections and disconnection are present. The nodes belonging to the WSNs have limited resources, are prone to failure, have limited memory and processing capacity and usually run on batteries. These features make the network performance a very important aspect when we need to compare different routing protocols.

The work in [15] presents and analyzes key performance metrics that adequately evaluate WSNs in real time. The networks are deployed in different scenarios, most of them in extreme or demanding conditions. As a consequence, the quality of service, QoS, must be maintained over time, and the performance metrics are critical parameters in assessing the network. The authors propose a taxonomy performance where parameters, such as delay tolerance, capacity, reliability, energy efficiency and fault tolerance, are established. These metrics are monitored in order to measure and maintain controlled levels of performance parameters.

Previous works show the IEEE 802.15.4 standard as a suitable solution to bottlenecks problems in WSNs, specifically at the MAC layer level. The authors in [16], for example, conducted a study of performance metrics, such as throughput, packet delivery rate and end-to-end delay under a static scenario. The influence of these parameters on energy consumption is investigated. Two commonly used performance parameters in WSNs are energy and delay [17]. The end-to-end delay is an important metric that depends on media access techniques; thus, collisions and retransmissions directly affect delay and, therefore, network performance. A specific technique presented in [18] analyzes metrics, such as packet loss, delay and energy consumption, to quantify the quality of service with the aim of improving the capacity of the network. They explain a traffic control scheme effective at achieving an adequate topology management. Maintaining reliable and optimal routes will decrease packet loss. Here, energy consumption in the network plays an important role, because if the batteries of some nodes are depleted, the network topology will change, and the remaining nodes must be able to reconfigure the links rapidly in order to avoid packet loss. In [19], cost metrics are measured based on link quality for various link configurations that influence energy and topology efficiency. The authors analyze metrics, such as packet success rate, signal-to-noise ratio and power, in order to propose an efficient combined metric.

A reliable routing protocol in WSNs is essential due to the versatility of these networks. In [20], the authors analyze metrics, such as end-to-end path reliability and the number of hops. Their work analyzes different routing algorithms based on link reliability models for each type of node. In [21], a routing protocol that guarantees the route with the shortest path while maintaining quality of service is designed. The route optimization is related to the ideal relay node position, and metrics, such as mean end-to-end delay and packet rate, under random scenarios are considered.

A scenario with a single collector node is considered in [22]. For this topology, the authors calculate the delay and the probability to form routes in the routing tables. This allows costs to be assigned to the links depending on energy consumption, so that they achieve the network topology adaptations dynamically according to the energy of the nodes with reliable packet transmission. Moreover, Busse *et al.* [23] considers many-to-one communication and analyzes the efficiency in forwarding packets as a function, not only of the hop count, but also of the link quality. This is reflected in lower energy consumption, due to a reduction in the packet loss. In addition, the influence of packet retransmissions in communication and its effects on energy efficiency in the network are analyzed. All factors related to the occupancy of the channel in wireless communications influence the multiple-hop networks and affect the network performance. Some of the most adaptable protocols to this type of network are AODV and DSR, which aim at reducing cost and energy consumption and improving reliability. These protocols allow multi-hopping among the actively involved nodes that want to establish and maintain routes in a network [24]. On the other hand, ZTR, a widely referenced algorithm, has low overhead and is simple with regard to the memory capacity of the nodes, since they do not have routing tables, which eliminates path searching and updating. Nevertheless, it has some drawbacks in terms of flexibility and adaptation, especially when it is deployed in wide network environments [25].

Regarding the energy problem, Gagarin *et al.* in [26], introduce a method for balancing costs with a clustered spanning hierarchical algorithm and an energy model based on transmission energy. In addition, they assigned weights to the links in a graph model and explain how the algorithm provides the best routes of the hierarchical tree. Self-organization of these distributed algorithms maximizes sensors lifetime. On the other hand, related to the power balance in the network, [27] treats the issue of energy distribution among the nodes as a function of the traffic load. In that paper, the authors set coronas around the sink node with the purpose of showing that the nodes closest to the sink carry more traffic load and, thus, spend energy more rapidly. Then, they propose as a solution the non-uniform distribution of the nodes, so that energy depletion is balanced. Along the same topic, the authors in [28] complement the analysis with the study of multi-hop routing and its impact on node distribution contrasted with direct the transmission mode and its effects on node distribution. They also address the problem through a topology of coronas around the sink node and analyze the inter-corona and intra-corona energies. Cuomo *et al.* in [29], study the network formation process and the topology focused on energy consumption. They vary factors, such as the number of sink nodes and their distribution in the network. Relationships between the number of sink nodes and energy consumption are established, and static and mobile scenarios are studied.

The work in [30] introduces the relationship of energy with the performance of the network. This problem is greater in networks with static nodes. The nodes closest to the root node or collector node are likely to deplete their energy first, because they generate traffic and also carry traffic from other network nodes. This difficulty is known as the depletion energy sink-hole problem. This can cause connectivity problems between the root node and the rest of the network, completely missing information at the destination. Tunca *et al.* [31] also tackle the energy reduction problem of the nodes nearer to the collector node. The authors propose a solution using mobile sinks. This balance solution works due to the topology changing, and the nodes are sometimes away from and sometimes near of the collector. However, this will affect overhead and delay, and it will improve the network performance, but traffic and energy are balanced, representing an important advantage. Moreover, metrics, such as energy, latency and reliability for the mobile sink routing protocol, are discussed.

## 3. Efficient Multi-Parent Hierarchical Routing Protocol

We have proposed a routing protocol that creates a logical hierarchical network topology, where the node hierarchy is given by its location level in the hierarchy (the lower the level, the lower the hierarchy) [13]; basically, the root hierarchy (coordinator node) will have the highest hierarchy. When a node is in a specific hierarchical level (or generation), the nodes that are connected to it and have a higher hierarchical level are parent nodes. On the other hand, nodes that are connected to any parent node and have a lower hierarchical level are child nodes. Thus, we named our proposal the multi-parent hierarchical (MPH) protocol. The hierarchical topology will be formed with routes that minimize the number of hops from a given node to the coordinator; this restriction will reduce the overall energy consumption of the network. The MPH routing protocol allows a child node to have one or more parents. As a result, a node can share both children and parents with another node belonging to the same hierarchical level or generation.

The coordinator might also send packets to nodes in the network. We adopt a source-routing approach for traffic to be sent from the coordinator to a node, because the coordinator node has more resources and capacity than the rest of the nodes. This means that it can easily gather information from the network, such as data generated by sensors, links quality metrics, neighborhood tables and other variables, that can be used for route finding, performance analysis and network optimization.

MPH protocol operates as a hierarchical tree: the nodes establish parent and child links that constitute the possible routes. Node hierarchies are used to establish parent-child links based on radio coverage. The coordinator node is the root node, and all nodes send information to that destination. If a node wants to send a packet to the destination node, it looks for its parents in its neighbor table, chooses a parent and sends the packet. This process is repeated until the packet reaches its final destination. The coordinator node knows the entire network topology through inquiry packets that it sends from time to time. They are passed through the hierarchical branches and, in their way, ask each node to send its information to the coordinator node. Thus, the coordinator node has valuable information of all nodes of the network, such as statistics, position, routing tables, and so on.

The MPH protocol has the advantage of rapid self-configuring links under node connections and disconnections. The node hierarchies are updated based on the highest hierarchy among directly-connected neighbors reestablishing parent-child relationships. Thus, MPH presents some mechanisms aimed at optimizing the communications among nodes and the coordinator: (1) The neighbor table maintaining process is originated by a *ND*(Neighbor Discovery) packet transmission. The sending and receiving nodes can update their neighbor tables with the other node address, once each node receives the corresponding acknowledgment. *ND* packets are delivered at regular specified intervals, so that nodes rediscover their neighbors at these intervals. The response to a *ND* packet is called *NDR*(Neighbor Discovery Response) and the response to a *NDR* packet is another packet, called *NDRACK* (Neighbor Discovery Response ACK). Both the *NDR* and *NDRACK* packets contain node hierarchy information; (2) Each time a node is involved in a neighbor table maintenance process, it should check if it is going to maintain or change its hierarchy. The rule is that the node selects as parents the set of neighbor nodes with the highest hierarchy among the registered nodes in its neighbor table and assigns itself a hierarchy that is one unit lower than his parent node(s). If a node has no neighbors or all neighbors have zero hierarchy, it will have zero hierarchy, too. This situation can occur in the network initialization transient stage when there are nodes that still have zero hierarchy; (3) A node updates its neighbors, and possibly its hierarchy, at regularly specified intervals. Thus, the neighbor discovering process, sending an *ND* packet, is always done not long after a possible topology change. The purpose of this mechanism is to reduce the time in which nodes update their hierarchies, especially when extreme conditions occur due to the loss of critical links in the logical topology. This will diminish auto-configuration network delays and reduce the packet loss problem caused by the disconnection of links; (4) In order to prevent unreliable changes, caused by node faults, MPH uses a scheme that employs a slow erasure of nodes from the neighbor table, for which purpose, a variable called neighbor persistence, is used. If an NDRpacket is received from a neighbor as a response to an *ND* packet, and persistence takes a maximum value *p*. Afterwards, prior to the transmission of an *ND* packet, persistence is reduced by one for every registered node in the neighbor table. Therefore, if after successive *p ND* packet emissions the origin node did not receive any *NDR* packets from the node that initially had persistence *p*, then the route to this node is erased from the table. This mechanism solves the problem of inconstant changes in the neighbor tables, which can be caused by the momentary disconnection of the nodes. For example, occasionally, due to interference or packet collisions, the nodes might not respond in time to their neighbors, which, without the node persistence mechanism, would modify the neighbor tables. Thus, this mechanism gives more opportunities for weak links to keep an association with the routing tables and avoids abrupt changes; (5) The coordinator node builds the whole topology of the network after it gathers the neighbor tables from the nodes. Then, the coordinator node is able to specify the complete path to be followed by the packet to reach its destination, and it has the capacity to ask for statistics of the nodes in the network [13].

**Figure 1.**
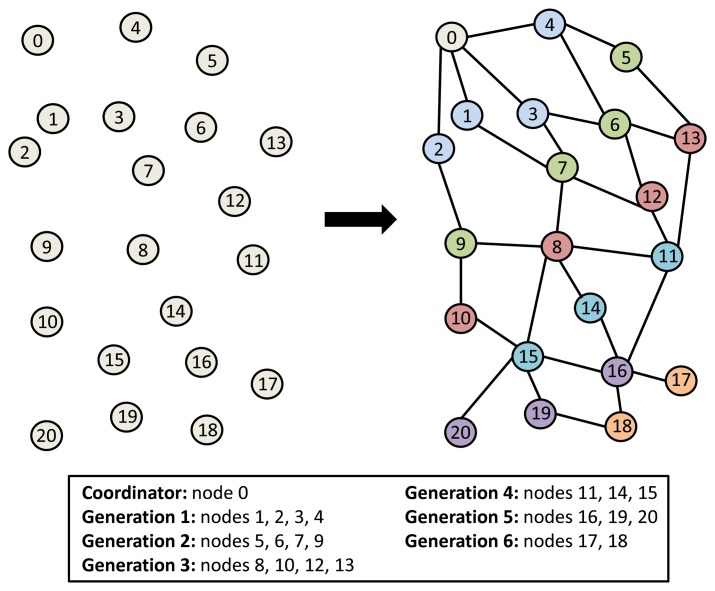
Multi-parent hierarchical (MPH) link formation process.

Figure 1 describes the link formation process followed to assign hierarchies to the nodes, so forming node generations. For the network formation process, each node establishes connections with other nodes within its coverage radio; these nodes are its neighbors and will belong to its neighbor table. Starting from the coordinator node, which is the node that has the highest hierarchy level, the nodes connected to it are assigned a lower hierarchy level (one level below the coordinator’s hierarchy). Then, the next lower hierarchy is assigned to the nodes connected to the second highest hierarchy level nodes, and so on, until the hierarchical topology is established. For a given node, among the nodes in its neighbor table, those with higher hierarchy are parent nodes, and those with lower hierarchy are child nodes. Nodes having the same hierarchy level will belong to the same generation.

### 3.1. Network Implementation under the MPH Protocol

**Figure 2.**
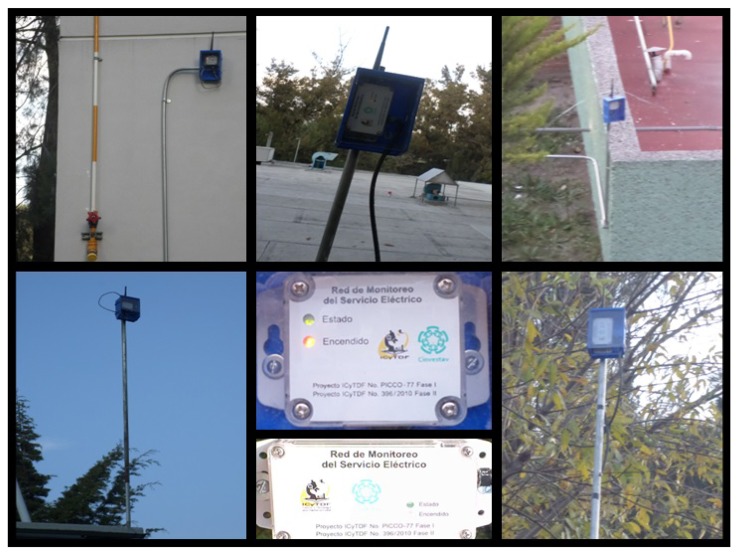
Wireless nodes.

The MPH protocol was successfully implemented in a real network under a variety of conditions of temperature, humidity and obstacles. For the purpose of validating our simulation tool, we compared the simulation results with experimental data collected from this network under the same conditions and parameters. The number of nodes forming the network was 98 plus one coordinator node, and the coverage area was 0.16 km^2^. Nodes 1 to 39 correspond to source nodes, which are nodes that generate traffic to the coordinator node, while Nodes 40 to 99 are relay nodes that simply pass traffic to the coordinator node, but do not generate traffic. We built the MPH protocol on top of the MAC layer of the IEEE802.15.4 standard using the CC2530 chip from Texas Instruments [32]. The power transmission of each node was 4.5 dBm. Figure 2 shows the nodes running the MPH protocol.

**Figure 3.**
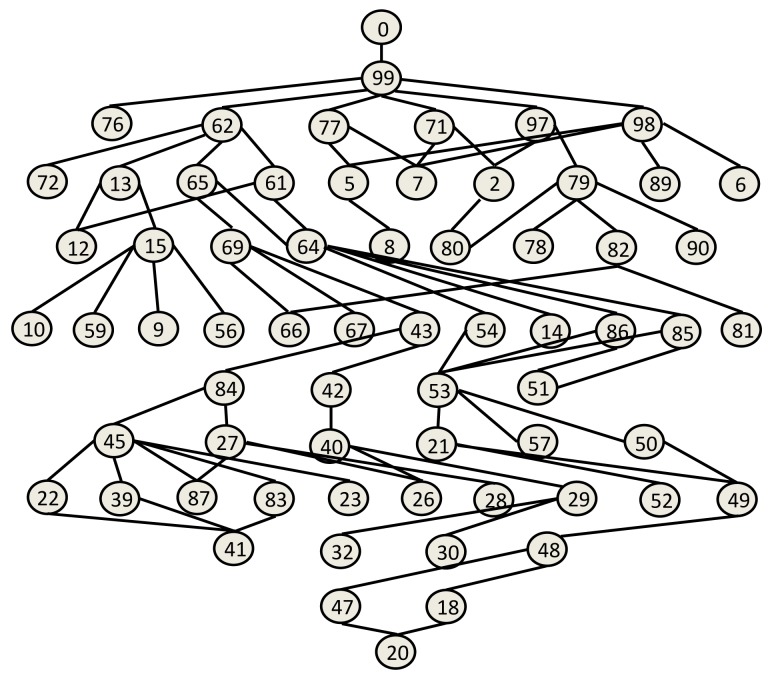
Real network.

Figure 3 shows the logical connections of the real network made with nodes built by us to test the MPH protocol. This same topology was used in the simulation to carry out the comparison. Table 1 shows the main Physical, MAC and Network layer parameters, which were used in the simulator and in the real network. We evaluated the performance of the MPH protocol of both the real network and the simulator in order to see the similarity of the results. We analyzed the average number of hops, the delay and the number of tries that it takes the coordinator to reach a node in the topology.

**Figure 4.**
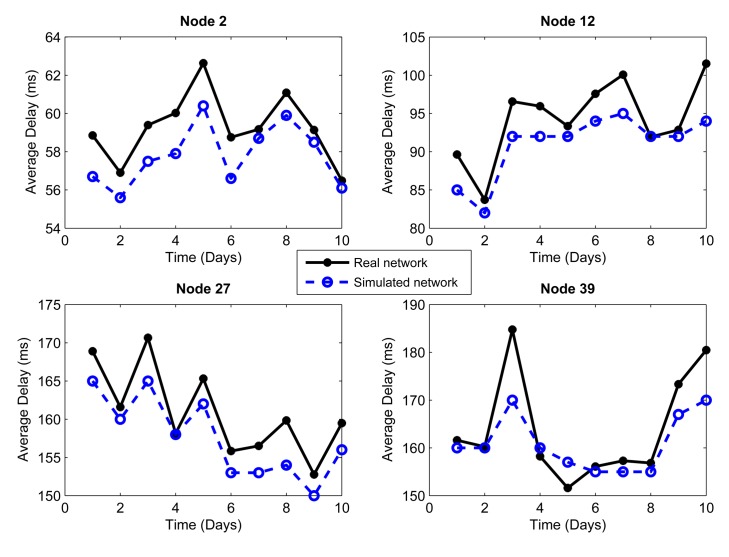
Average delay per day in source nodes.

**Figure 5.**
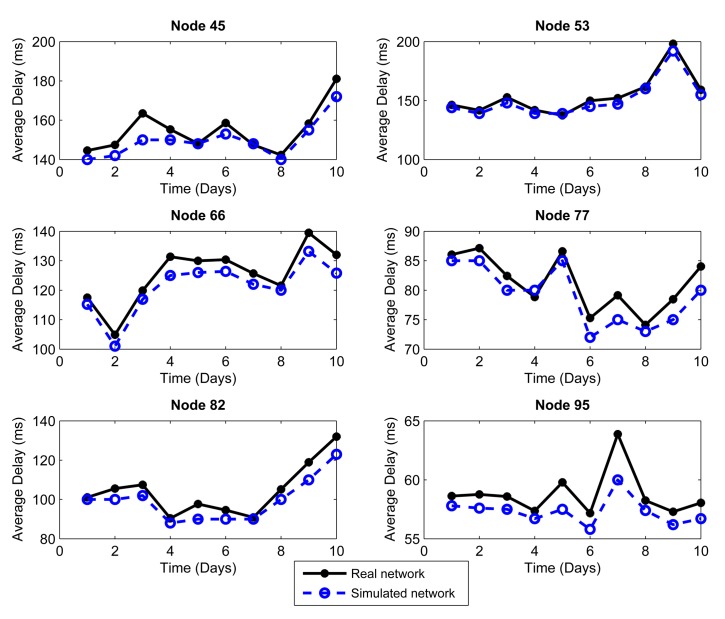
Average delay per day in relay nodes.

**Figure 6.**
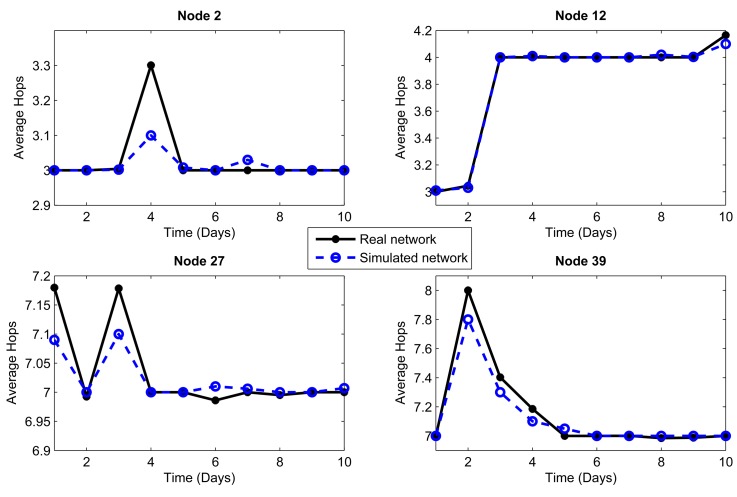
Average hops per day in source nodes.

**Figure 7.**
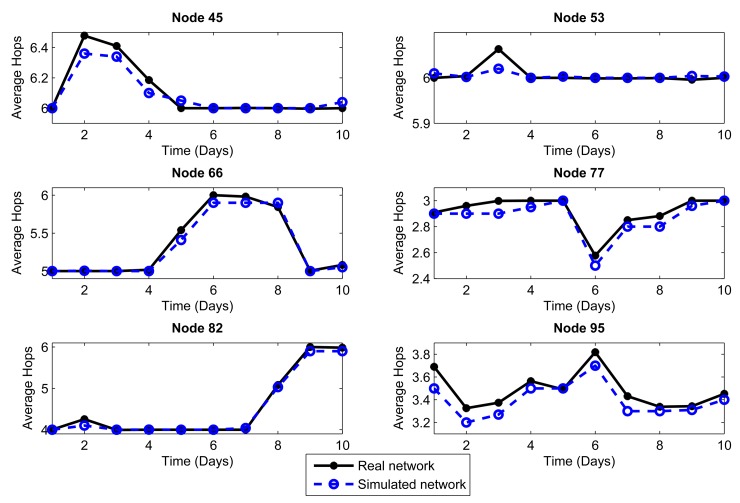
Average hops per day in relay nodes.

**Figure 8.**
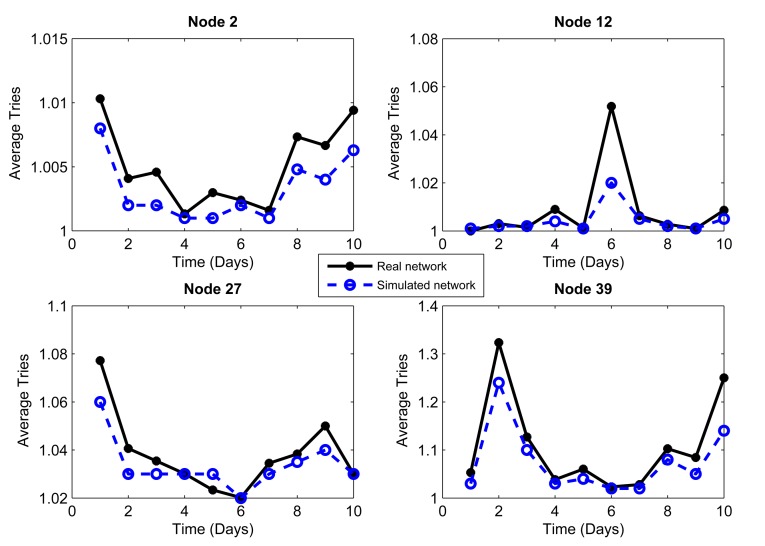
Average number of tries per day in source nodes.

**Figure 9.**
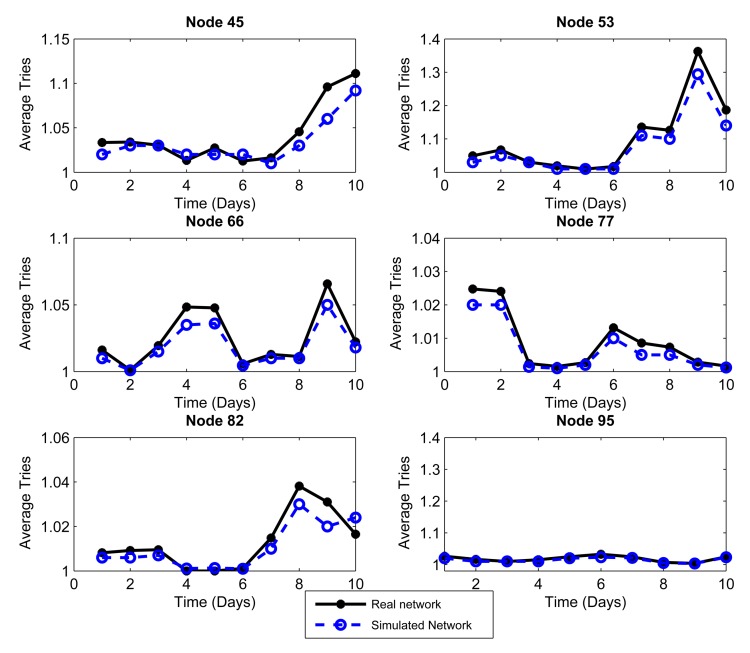
Average number of tries per day in relay nodes.

We considered the performance of the network for ten days of continuous operation of the nodes. For this reason, we must consider that the topology of Figure 3 is the initial topology, and this may change due to environmental conditions, such as interference and bad channel behavior. In our simulator, the conditions of each day were represented by changing physical layer parameters, such as packet loss, which includes factors, such as interference, bad channel conditions and fading. Figures 4, 5, 6, 7, 8 and 9 show the results.

The results are generated for the average delay, number of tries and number of hops per day. We chose 10 representative nodes of the 99 nodes that simulated the network. Among the source nodes, we chose randomly Nodes 2, 12, 27 and 39, while among the relay nodes, we chose randomly Nodes 45, 53, 66, 77, 82 and 95. In this way, we can give a proper and close approach in order to measure the reliability that our simulator has against a real scenario under the same conditions.

The conditions of interference, attenuation and other environmental variations can be represented in the simulator as a percentage of packet loss. Due to connections and disconnections of the nodes, we can observe time periods when the network behavior is not considered as normal, and we can assume special conditions for these periods. Considering the standard deviation of the packet loss each day, we can make an estimate of the network behavior. We analyzed the relationship between the condition of the links on the topology and the packet loss, and we empirically established intervals. If the environment affects the network behavior with less impact, the percentage of packet loss will vary between 1% and 2%. If the impact on the network performance is greater, the packet loss rate will vary between 2% and 5%. This percentage of packet loss will be reflected in the network delays and in the momentary connections and disconnections of nodes: it will therefore affect the number of hops. We took into account the average delay as a parameter to consider one or the other packet loss interval. Thus, we obtained a mean value of the delay for the ten day interval; for the days with delay above the mean, a packet loss in the interval (2%–5%] was used, whereas for those with delay below the mean, a packet loss in the interval [1%–2%] was used.

Figures 4 and 5 show, respectively, the mean delay per packet averaged over a day for the selected relay and source nodes, considering a monitoring time of each node every 30 s in a day, approximately. The continuous lines show the performance of the real network described before, whereas the dotted lines show the simulated network using the C++ simulator constructed by us. Here, we can see that the delay depends on the channel conditions, and not every day had the same environmental conditions.

Another important metric that clearly shows the connections and disconnections of the nodes during the operation of the network is the number of hops, also called hop count. Figures 6 and 7 show the mean number of hops per packet averaged over a day for each of the nodes (source and relay, respectively) that were chosen as examples. The number of hops will directly impact the packet delivery delay, because if a node is disconnected, the packet route will change, perhaps making it slightly longer and requiring more time to reach the coordinator node. Both metrics, the number of hops and delay, also affect the energy consumption, because the more the node disconnects, the more the number of hops to the destination and the greater the packet delay. Here, we observe that the simulated network has a behavior very close to the real network. Therefore, the physical layer parameters assumed, from one day that extreme environmental conditions took place, reflect quite well the behavior of the network.

The coordinator node is a robust node, with more processing resources than the other nodes. Consequently, this node has the ability to process all of the statistical information collected from the network nodes. The coordinator has a source routing mechanism [13] to request information from the nodes. This information can be statistical processes, neighbor tables, sensing measurements and all of the features that the coordinator node needs to know to be aware of the nodes that form the network topology. When the coordinator needs to communicate with a network node, it sends a packet to that node, and this is called a “try”. The mean number of tries is a metric that reflects the number of times that the coordinator attempts to connect to a node before an ACK is received. Figures 8 and 9 show the average number of tries per packet averaged over a day for 10 nodes chosen as the example. Figures 8 and 9 show that the source routing mechanism programmed into the simulator is quite similar and has a performance comparable to the mechanism of the real network.

We see that the simulator displays a trend close to the behavior of each node in the network, which shows that we have a reliable computational approach with respect to a network in a real-life environment. We confirm this statement by calculating the mean relative error of the measurements obtained by the simulation with respect to the measurements given by the real network. For the delay, the simulator has an error of 2.97%; for the number of hops, the simulator has a 0.87% error; and for the number of tries, the simulator has an error of 1.08%.

## 4. Proposed Metrics

In this section, we compare the performance of the MPH protocol with that of commonly-used protocols in sensor networks, such as AODV, DSR and the well-known algorithm, ZTR. Although AODV and DSR are reactive protocols and ZTR and MPH have a hierarchical routing configuration, we can measure their performance based on the specific metrics presented below and then compare the efficiency of each protocol regarding overall energy consumption. We use metrics, such as retransmissions, retries of the CSMA/CA algorithm when the channel is busy, overhead, discovered and valid routes and energy at the network. We analyze these metrics and observe their impact on problems, such as re-configuration, node damage, interference and energy consumption.

Regarding the channel, the CSMA/CA (carrier sense multiple access with collision avoidance) protocol works such that a node listens to the channel, and, if it is free, the node transmits; if the channel is busy, the node recalculates a random delay for which it waits in order to listen again. This metric is what we call CSMA/CA retries. If these CSMA/CA retries happen many times, this means that the channel is busy most of the time; possibly much overhead will cause many collisions. Moreover, when the channel is constantly busy, there are several collisions and packet retransmissions. This action highly influences the energy demands, because nodes decrease their capacity if they are continually retransmitting the same packet. Therefore, if a node has to retransmit a packet many times, all of these retransmissions can degrade the network performance, increasing the number of collisions and the energy consumption.

In the network layer, overhead is an important factor in energy consumption. The routing protocol efficiency can also be measured by the number of packets needed by the protocol to route information to the destination. A protocol with many control packets will contribute to packet collisions and a reduction of the throughput. Regarding discovered routes, in all considered protocols, nodes have the capacity to know their neighbors. Under AODV and DSR, nodes update the routes when they are required. In contrast, under ZTR and MPH, nodes update the routes on a periodic time basis. Knowing the neighbors helps nodes to have valid routes to the destination, to correctly forward packets and to decrease the number of retransmissions needed. Valid routes are routes that are not corrupted, and are available for immediate use. If under a routing protocol nodes can handle several valid routes, packets will have more chances to be forwarded, will not be continually retransmitted and energy resources will be saved.

The energy consumption in a network is directly related to the complexity in managing routing or neighbor tables. As the sensors carry out many routing algorithms and processes, for example, to choose the best route among many available in the routing tables, they will increase the energy consumption.

### 4.1. Simulation Environment

**Figure 10.**
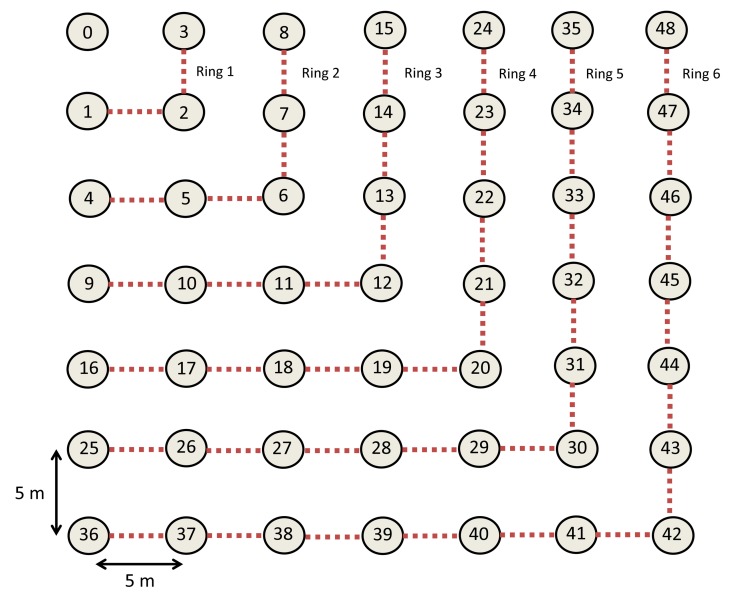
Network topology.

One aim of this work is to define important metrics that influence energy consumption in a WSN. In order to assess the performance of the mentioned protocols, we use the same simulator that was validated in Section 2 with the MPH protocol and a real network. For the simulations, we use a regular topology with a well-defined arrangement, with the purpose of having a more exact reproduction and having more accuracy when measurements are collected. Thus, in the simulations, we used a grid topology of 49 nodes; each node has a separation distance of 5 m and a coverage range of 8 m. The coordinator node is located in a corner, establishing crowns or rings of nodes around the root node, as shown in Figure 10.

The advantage of using a topology comprised of rings around the coordinator node is that it allows one to analyze groups of nodes with similar features. The nodes belonging to the same ring carry approximately the same traffic, from themselves or from other nodes, and hence, they have approximately similar energy consumption. In this way, we can make both global and local topology analysis.

All network nodes must forward packets to the coordinator node directly (one hop) or indirectly (multiple hops, forming a route with intermediate nodes). It is clear that nodes closer to the coordinator node will carry a larger number of packets than the nodes farther away and this will produce an unbalance in the energy depleted in different parts of the network. For example, nodes in the first ring in Figure 10 (Nodes 1, 2 and 3) are the only ones with a direct link to the coordinator, and all other nodes must send packets through these nodes, making them deplete their batteries more rapidly.

A summary of the features of the simulation scenario can be seen in Table 2. Nodes generate packets with inter-arrival times given by a random variable *T* with exponential distribution with parameter *λ*, as in Equation (1):

(1) $$F_{T}(t)=P(T\leq t)=1-e^{-\lambda t},t\geq 0$$

where *λ* represents the average number of arrivals per time unit. Thus, packet arrivals follow a Poisson distribution.

In the AODV routing protocol, route search is done as follows. A node sends a RREQ packet (route request) in broadcast mode in order to apply for a route, indicating the destination node, the source node identifier, hop count initialized to zero and its sequence number. If a node receives this packet, it first checks if it has previously received a record of this RREQ packet. If the packet is not registered, the node retransmits it again, increasing the number of hops and creating a reverse path (the path to the node that this RREQ has reached). A node that has received an RREQ packet can respond with an RREP packet (route reply) to confirm a route in only two cases: if the node is already the destination or if the node has an available route to the destination. When a node responds with an RREP, this packet is sent, with the corresponding path, in unicast mode to the neighbor who had sent the RREQ. This procedure continues until the source is reached; at this point, the route has been formed. The source node may receive RREP packets from different nodes as a confirmation of various possible routes to the destination. In this case, the source node has two criteria for selecting the best route: the route that has the least number of hops or the route that has the highest sequence number. In this way, in the AODV routing protocol, nodes can have multiple routes to the same destination; the node generally chooses the shortest route (the route with fewer hops).

The route search of the DSR routing protocol is as follows. When a source node has a packet to send to a destination, it first looks in its cache to see if it has a route to that destination; if so, it creates a new packet header containing the path with the necessary hops to reach the destination and sends it. Normally, the source obtains the route by searching in its cache for routes previously discovered. If there is no route to that destination in its cache, it is time to start the route discovery mechanism to dynamically find a new route to the destination node. First, the source node sends an RREQ broadcast packet, which contains the identifier of the source node and that of the destination node, whose route is to be discovered; it also contains a unique identifier for the RREQ. Each intermediate node makes a copy of the received RREQ and adds its identifier. When a node receives an RREQ, it first looks in its cache to find out if it has the route to the destination node. If so, the node responds with an RREP to the source instead of forwarding the RREQ. In that RREP, the node writes all of the nodes that the received RREQ has passed through and the nodes of the route found in its cache. If the node does not find a route in its cache, it adds its address in the packet and forwards it via broadcast. Once the source node receives the RREP, it stores the route in its cache. This route will be included in the header of each subsequent packet that the node sends, so that all nodes that receive the packet will know to which next node they should resend. As with AODV, nodes can have multiple routes to a destination and choose, for example, those with the lowest hop count.

Routing with ZTR is established based on parent-child links. The coordinator node is responsible for creating the network configuration, choosing the parameters and setting the maximum depth of the tree network. Nodes maintain their address information from their parent and child nodes. To send a packet in this rooted tree scheme, a node observes, based on the destination address, if it is a downward path; if so, it sends it to one of its children; otherwise, the node sends it to its parent [1].

In order to evaluate the protocols performance, the authors use an event-driven network simulator with 1% of packet loss in all the physical layer links. The MAC layer is constructed under the mechanism of non-persistent unslotted CSMA/CA [33], established by the IEEE 802.15.4/ZigBee standard, with the collision avoidance model and retransmissions. The network layer was tested with routing protocol implementations, such as AODV, DSR, the ZTR algorithm and our own protocol, MPH.

### 4.2. Results and Discussion of the Proposed Metrics

The metrics discussed in this section and used in our evaluations are important to study the network performance. The analysis of these metrics is intended to give a measure of quality in the delivery of information in the network. The particular parameters of robustness that have to be taken into account depend on the type of application of the network. For example, if a network is designed to be deployed in a large area, it is essential that the number of hops and the interference of the environment be measured rigorously and that the channel conditions be observed closely. Likewise, if a network is to be implemented in an indoor or outdoor environment, it is essential to know the roll that the routing protocol will play. Now, we classify the metrics of interest into three categories: MAC layer metrics, network layer metrics and a global metric.

#### 4.2.1. MAC Layer Metrics

MAC layer metrics reflect channel occupancy and throughput, which are parameters that have a direct impact on the use of bandwidth and the availability of the nodes to send and receive packets. Furthermore, the number of collisions in a network is a parameter that is reflected in the number of packet retransmissions, which gives an indication of interference in the channel, malfunctioning nodes or attacks.

##### Retransmissions and CSMA/CA Retries

As said before, the MAC layer protocol implemented for the three protocols studied here is non-persistent CSMA/CA. Regarding the retransmissions, the MAC layer protocol implemented has a maximum of three retransmissions for each packet. If a packet has more than three collisions, the node will not replicate the packet anymore and the packet will be dropped. Therefore, when the average number of retransmissions is very close to three, this means that the channel is heavily congested, and therefore, there are many collisions.

Before transmitting a packet, the CSMA/CA algorithm calculates a delay of (2*^BE^* − 1) backoff units, where *BE* is the backoff exponent, and the node waits for this delay. Then, the node performs the clear channel assessment (CCA) to listen to the channel. If the channel is free, the node transmits; if the channel is busy, the node increments the value of *NB* by one, where *NB* is the number of times the CSMA/CA algorithm was required to backoff in its attempt to transmit (*NB* starts at zero) and applies Equation (2):

(2) BE=min(BE+1,macMaxBE)

where *macMaxBE* is the maximum value of the backoff exponent, which for the CSMA/CA standard is five. Subsequently, if:

(3) NB>macMaxCSMABackoffs

is true, the packet is dropped. If it is false, the algorithm recalculates the delay again. Here, *macMaxCSMABackoffs* is the maximum number of backoffs that the CSMA/CA algorithm will attempt before dropping the packet [33]. Therefore, when the average number of CSMA/CA retries is very close to five, this means that the channel is constantly busy and nodes should wait longer to transmit. This metric directly influences the network delay.

**Figure 11.**
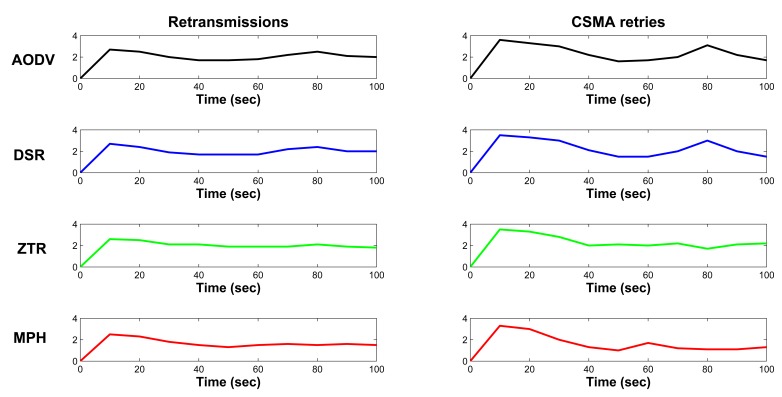
Average retransmissions and CSMA/CA retries vs. time (s).

Figure 11 displays the average node retransmissions and the average CSMA/CA retries for the four studied protocols. Here, we remark that for the four protocols, initially, there are many retransmissions and CSMA/CA retries. This is so because when the nodes connect to the network, they begin by sending broadcast packets to discover neighbors, so there is a greater number of packets in the network: overhead and traffic packets produce more collisions. Note that in the retransmissions, during the first 10 s, the four protocols increase their average (the line has the highest peak of the graph). This is because of the amount of packets flowing in the network during the time of the formation of the topology. The CSMA retries also show this peak, because as the channel is constantly busy, the CSMA retries increase. However, regarding retransmissions, it is important to mention that this first peak has an average value of 2.7 for AODV and DSR, 2.6 for ZTR and 2.5 for MPH. Concerning also the first peak, the CSMA retry metric has an average value of 3.6 for AODV, 3.5 for DSR and ZTR and 3.3 for MPH.

During the following 60 s, the protocols maintain a stability period. Regarding retransmissions, this period has an average of 1.98 for AODV, 1.93 for DSR, 2.1 for ZTR and 1.7 for MPH. As regards CSMA retries, this period has an average of 2.3 for AODV, 2.2 for DSR, 2.4 for ZTR and 1.7 for MPH. Note that after approximately 60 s, the AODV and DSR protocols increase retransmissions and CSMA/CA retries again; this is possibly due to the onset of expired routes and routes that are no longer valid. Consequently, nodes must re-learn some routes, and the overhead increases again. However, the MPH protocol does not exhibit this latter peak, because nodes periodically reconfirm their neighbors, and the information in their neighbor tables is reliable, as it is constantly updated (due to the fact that MPH is a proactive protocol). Besides, MPH allows any child node to have several parents, so if an upward route is lost, there may be other backup routes that can be used, so preventing packet retransmissions. ZTR has the largest number of retransmissions and CSMA/CA retries of all of the protocols, because when a node loses connection with their neighbors, all of the child nodes remain without communication to send packets to the coordinator. Therefore, the nodes attempt to retransmit packets; this may also cause the channel to be busy for more time, and the CSMA/CA retries to become higher. ZTR shows no peak, because there is no route expiration. Approximately at 80 s, AODV and DSR have a new retransmissions peak value of 2.5 and 2.4 respectively. Moreover, there are also peak values of 3.1 for AODV and three for DSR regarding CSMA retries. Therefore, we can establish that the MPH protocol shows a reduction on retransmissions of 19.3%, 17.4% and 17.8% compared to AODV, DSR and ZTR respectively. Furthermore, MPH presents a reduction on CSMA/CA retries of 30.3%, 27.3% and 28.9% with respect to AODV, DSR and ZTR correspondingly.

#### 4.2.2. Network Layer Metrics

The metrics of the network layer are very important, because they show the performance and usefulness of a routing protocol. Each routing protocol is designed for specific applications and certain scenarios. These metrics indicate how the use of bandwidth is affected by the overhead of the routing protocol in use. In addition, the availability of effective routes and the ability of the network for self-configuration show the capacity of the protocol to recover from topology changes. Recovery times have an impact on the latency in the network.

##### Overhead

Overhead is an important metric that influences the amount of network collisions and the channel occupancy, and this is why we compare this metric for each of the protocols under study. Overhead depends on the number of routing packets that the routing protocol needs to connect to nodes and to route packet traffic. Thus, it is calculated taking into account the number of control packets that are needed to route a traffic packet. Ideally, a routing protocol should need the least amount of control packets.

**Figure 12.**
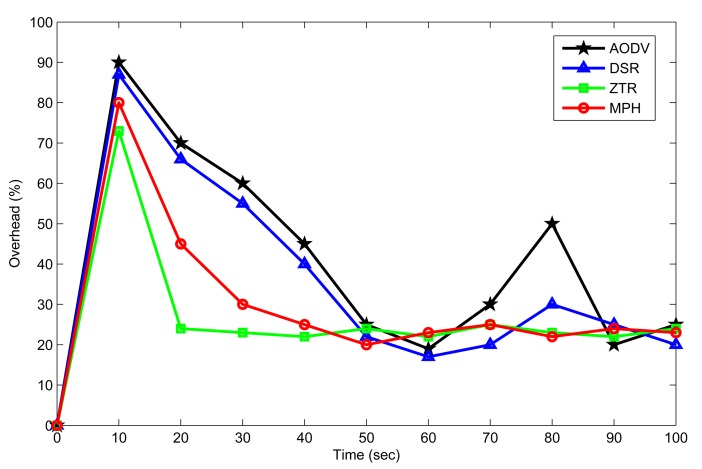
The percent of overhead vs. time.

Overhead as a function of time is presented for each of the protocols in Figure 12. Overhead represents the fraction of the total transmitted packets that are control packets. At the beginning of the simulation, the nodes know the network through their neighbors, so as a part of the routing protocol, the nodes send discovery and response packets. For this reason, there is a more pronounced peak at the beginning of the plot. Subsequently, AODV and DSR protocols send route discovery packets periodically when a node does not have a route to a desired destination, because this has expired or is broken. The AODV protocol has expiration timers for routes, so it has more overhead than the others. DSR does not have timers, and it only sends error messages for invalid routes. ZTR is the protocol with the least overhead, since it does not keep routing tables in the nodes, as routing is assigned by the coordinator at the beginning. The MPH protocol presents a behavior similar to ZTR, even though MPH has neighbor discovery packets to form the neighbor tables, response neighbor discovery packets and a verification reply ACK packet. This shows that MPH does not congest the network with overhead packets and can be similar in behavior to the simple protocol, ZTR. We can see that MPH protocol shows a reduction of 26.9% on overhead with respect to AODV and 17% with respect to DSR. Besides, MPH presents only a 11% increment on overhead with respect to ZTR.

##### Discovered Routes

The discovered routes percentage is a metric that measures how quick a routing protocol is at finding the routes needed to reach the coordinator node (because for this scenario, the coordinator node is the final destination). These routes may be direct links to the coordinator node or indirect links requiring intermediate nodes. This percentage is calculated considering the maximum number of possible routes that each node can reach in each of the evaluated protocols. This amount of routes is based basically on the average number of neighbors of each node, according to the information on the neighbor or routing tables. This metric reflects the time taken to find these routes: the faster the nodes find these routes, the sooner they will start sending information packets to the destination. This metric is also affected by the amount of maintenance routes that each of the routing protocols has.

**Figure 13.**
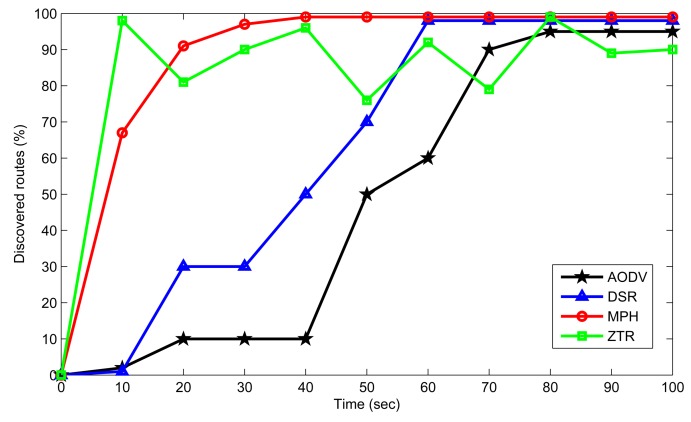
Percentage of discovered routes vs. time.

Discovered routes analysis is shown in Figure 13. MPH has already-defined routes when a node has a packet to send. This is why in Figure 13 we observe that MPH quickly reaches almost 100% of the routes to the destination node, whereas the AODV and DSR protocols take a little longer to find the routes due to the discovery mechanism and, if these routes have expired, to complete the whole discovery process. MPH checks the routes every determined time period, and this produces reliable routes throughout the operation of the network. On the other hand, we observe that ZTR shows some instability, because when a node loses connection momentarily, all of its child nodes are left without an ascending route. This means that these child nodes are left with no available route to the coordinator until a parent node reconnects to the network. For this reason, ZTR sometimes presents fewer available routes to the coordinator.

From the above discussion, it is worth pointing out the important fact that MPH is a proactive protocol, where routes are rediscovered on a periodic time basis, while AODV and DSR are reactive protocols, where routes are discovered when they are required. MPH has the advantage of handling neighbor tables instead of routing tables and has a quick and simple hierarchical routing algorithm. We can observe that at 100 s, MPH shows 43.1% more discovered routes than AODV, and 27.7% more discovered routes than DSR and ZTR, respectively. This means that MPH has the capacity to maintain more routes than the other protocols.

##### Valid Routes

A valid path is one that is effective at transporting packets. For AODV, a valid route is a route that has not expired and has not gotten an error packet called the route error (RERR), which reports that this route has been dropped. The same happens for DSR, except that it has no route expiration timers; in this case, a node knows that a route has become invalid, because it has received a route error packet. For MPH, the route validity is given by the persistence mechanism [13], which prevents unreliable changes in the neighbor tables caused by node faults. This scheme employs a slow erasure of a neighbor node based on the number of times that a node does not respond to a neighbor discovery packet.

In order to analyze the network behavior for the four mentioned protocols, we turned off at random different percentages of nodes, and 10 s later, we turned on these nodes. When we turn on the nodes again, we analyze the recovery time of the topology. As soon as the nodes are on, we start measuring the time it takes for each protocol to reestablish the valid routes to the destination, which is the coordinator node. This test was performed by comparing the tables of the nodes when the network showed normal behavior (with no node disconnections) with the tables of the nodes when the topology is restored.

**Figure 14.**
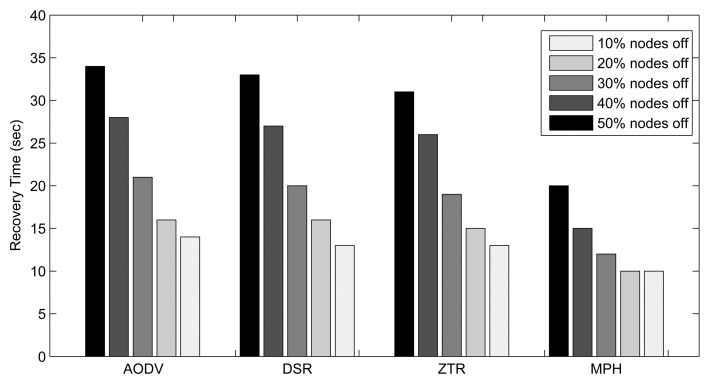
Recovery time depending on the percent of nodes off for the four protocols.

Simulation results are shown in Figure 14 through bar plots of the recovery time as a function of the percentage of nodes off for all protocols. As can be appreciated, MPH quickly reestablishes the routes based on the restored topology. AODV and DSR, on the other hand, reestablish routes when needed, and this process also depends on the packet error report, the invalid routes and the expiration timers. In ZTR, with the connections and disconnections of nodes, the network reorganization is set off when nodes start receiving error messages. When a node wants to re-connect to the network, it must re-acquire an address, and this occurs when a parent node accepts it as a child node. The exchange of messages required for this task results in somewhat more time consumption than for MPH reorganization. MPH uses the reactivity ND packet mechanism to quickly recover connections with nodes that have been reconnected to the network and the persistent nodes mechanism for maintaining reliable links to the neighbors [13]. Thus, for MPH, the recovery time for valid routes is lower. AODV and DSR use the route discovery in broadcast mode when the path to a new destination is needed and then have to wait until a node knows the path to the desired destination. We can determine in the worst case of 50% of nodes turned off that the recovery time of MPH is 58.8% that of AODV, 60.6% that of DSR and 64.5% that of ZTR

#### 4.2.3. Global Metric

Energy is a global metric that has an impact on autonomy in wireless networks. It is important that these networks operate for long periods without human intervention. All performance metrics mentioned in this work impact the energy consumption of the nodes directly or indirectly. This energy consumption is reflected in the life of the batteries and, therefore, in the connections and disconnections of nodes. All these topology changes represent more effort for the routing protocol, as they keep the channel busy while the network becomes reconfigured again.

##### Proposed Analytical Energy Model

A contribution of our work is our proposed energy model capable of quantifying the total energy consumed and the energy invested in the main activities of the nodes in a network. This model is based on the time it takes each node to perform the main tasks as a part of a network. This time is reflected as a consumed energy in each activity and is calculated depending on the parameters involved in each task performed by the node. These times and the voltage and current consumption are listed in Table 3, where the different types of energy considered are shown. Our work considers the energy spent by the MAC and the network layers. An analytical model based on the CC2530 chip operation, the system-on-a-chip (SoC) solution for IEEE 802.15.4, ZigBee, from Texas Instruments, is proposed here. This model describes the energy used for each of the functions performed by a CC2530 chip, from the moment that a node is on and is part of the network, as it receives and sends messages, runs MAC layer algorithms and changes of state, to the time the node is off.

This model includes the main types of energy of the basic activities that sensors carry out in a common performance in a network. These types of energy are: microcontroller energy (*E_MC_*), start-up energy (*E_Starting_*), shutdown energy (*E_Shutdown_*), CSMA/CA algorithm energy (*E_CSMA_*), switching energy (*E_Switching_*), transmission energy (*E_TX_*) and receiving energy (*E_RX_*). All of these energies are expressed in Joules.

**Figure 15.**
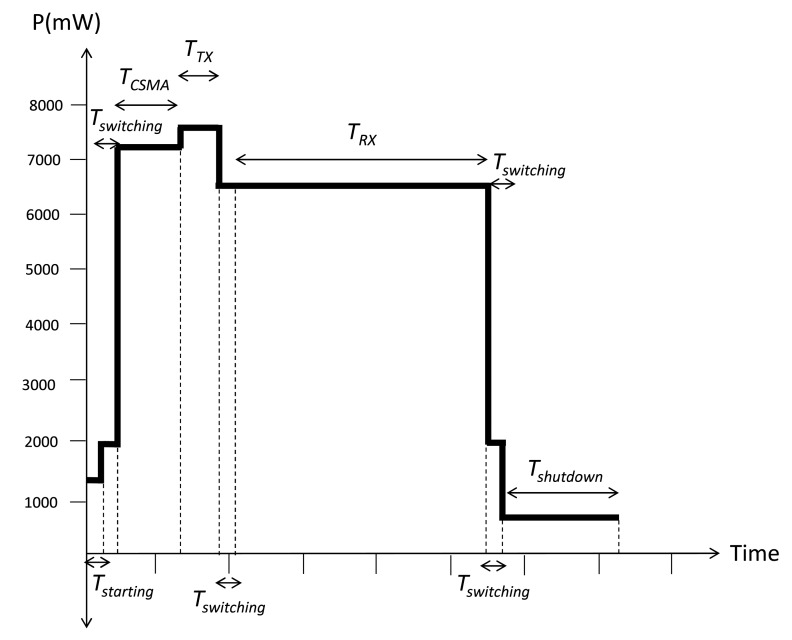
Conceptual scheme.

In order to better understand the process of using the various types of energy under the proposed model, Figure 15 explains this scheme conceptually. The process shown here is repeated several times during the sampling time, according to whether a node sends or receives packets. First, a node takes a starting time (*t_Starting_*) in order to turn on. Then, a switching time (*t_Switching_*) is taken to change the function before sending a packet to the medium. The node first runs the CSMA algorithm using a CSMA time (*t_CSMA_*). Now, the node transmits a packet using a transmission time (*t_TX_*). Again, the node takes a switching time (*t_Switching_*) in order to change activities, remains in the idle state (*t_Idle_*) and returns to change the task, taking a switching time (*t_Switching_*) to start receiving using a receiving time (*t_RX_*). The node will perform this chain of activities as many times as it sends and receives packets during the sampling period. Finally, the node turns off using a shutdown time (*t_Shutdown_*). All of the time, the microcontroller stays in active mode.

This process gives a measure of the energy spent for each of the principal activities of a node in the network. Depending on the task of a node and the time it takes to execute it, this corresponds to certain values of voltage and current; thus, we can obtain the total energy used by each node in each of the functions performed in the network based on the model above [34].

Microcontroller energy will depend on the node operation mode. For instance, sleeping techniques reduce energy consumption by setting the unit microcontroller into idle mode at given time intervals. Nevertheless, for our analysis, we assume that the CC2530 in each node operates in continuous active mode running at a 32-MHz clock frequency, since we want to study how the energy consumption behaves under a given routing protocol without the influence of sleeping techniques. Therefore, total microcontroller energy will be given by Equation (4):

(4) $$E_{MC}=T_{MC}\times I_{MC}\times V_{MC}$$

where *T_MC_* is the time in seconds in which the microcontroller unit operates at *V_MC_* volts and *I_MC_* amperes.

The start-up energy is calculated based on the voltage, current and time it takes the nodes to turn on and be ready for the network and is described in Equation (5):

(5) $$E_{\text{Starting}}=T_{\text{Starting}}\times I_{\text{Starting}}\times V_{\text{Starting}}$$

where *T_Starting_* is the time in seconds that it takes to turn on a node while it operates at *V_Starting_* volts and *I_Starting_* amperes.

The model also describes the energy spent by turning off nodes when the network time is over: this is the sampling period. This energy is given in Equation (6), and it is called shutdown energy.

(6) $$E_{\text{Shutdown}}=T_{\text{Shutdown}}\times I_{\text{Shutdown}}\times V_{\text{Shutdown}}$$

where *T_shutdown_* is the time in seconds that it takes to turn off a node while it operates at *V_Shutdown_* volts and *I_Shutdown_* amperes.

The switching energy is consumed when the node changes from the receiving mode to the transmitting mode or *vice versa*. It is given by Equation (7):

(7) $$E_{\text{Switching}}=T_{\text{Switching}}\times I_{\text{Switching}}\times V_{\text{Switching}}$$

where *T_Switching_* is the time in seconds it takes a node to switch from receiving to transmitting (or from transmitting to receiving) while it is driven by *V_Switching_* volts and *I_Switching_* amperes.

The CSMA/CA algorithm states that whenever a node wants to transmit, first it checks if the channel is idle. If it is, then it can transmit; otherwise, it must back off a random time prior to another transmission attempt. The energy consumed for the CSMA/CA algorithm is given by Equation (8):

(8) $$E_{\text{CSMA}}=T_{\text{CSMA}}\times I_{\text{CSMA}}\times V_{\text{CSMA}}$$

where *T_CSMA_* is the time in seconds during which a node runs the CSMA/CA algorithm while it operates at *V_CSMA_* volts and *I_CSMA_* amperes.

Transmission energy corresponds to the radio energy in transmission mode. In this case, nodes transmit packets from themselves or forward packets from other nodes. The transmission energy is described in Equation (9):

(9) $$E_{TX}=P_{\text{Length}}\times T_{TX}\times I_{TX}\times V_{TX}$$

where *P_Length_* is the length of a packet in bytes and *T_TX_* is the time in seconds it takes a node to send one byte while it operates at *V_TX_* volts and *I_TX_* amperes.

As in the transmission mode, a node consumes receiving energy when it receives a packet. This energy is shown in Equation (10):

(10) $$E_{RX}=P_{\text{Length}}\times T_{RX}\times I_{RX}\times V_{RX}$$

where *P_Length_* is the length of a packet in bytes and *T_RX_* is the time in seconds it takes a node to receive one byte while it operates at *V_RX_* volts and *I_RX_* amperes.

Values from Equations (4) to (10) are presented in Table 3. Therefore, the total energy can be calculated with Equation (11):

(11) $$E_{\text{Total}}=E_{MC}+E_{\text{Starting}}+E_{\text{Shutdown}}+E_{\text{CSMA}}+E_{\text{Switching}}+E_{TX}+E_{RX}$$

The energy model described by the above equations measures the energy used by the main functions of a node belonging to a sensor network, assuming that the functions related to the wireless communications are the most relevant from an energy point of view.

With this model, we analyze global and local energies. Let us name the energy of node *i* as *nodeEnergy_i_*, which is obtained by adding the energy spent by node *i* to perform each function in the network. For example, when a node connects to the network, it has zero energy consumed, so *nodeEnergy_i_* = 0.

In this energy model, we have energies that depend directly on the number of transmitted packets and other energies that do not. The energies related to the number of packets that a node carries are: *E_TX_, E_RX_, E_Switching_* and *E_CSMA_*. The energies that depend only on the node operation are: *E_Starting_, E_Shutdown_* and *E_MC_*. Thus,

(12) $$\text{nodeEnerg}y_{i}=\overset{\text{Packet dependent}}{\overset{︷}{E_{TX_{i}}+E_{RX_{i}}+E_{\text{Switchin}g_{i}}+E_{\text{CSM}A_{i}}}}+\overset{\text{Packet independent}}{\overset{︷}{E_{MC_{i}}+E_{\text{Startin}g_{i}}+E_{\text{Shutdow}n_{i}}}}$$

Now, in order to calculate each node energy, we are going to start with the packet dependent energies. *E_TXi_* will be the transmitted energy in node *i*, then,

(13) $$E_{TX_{i}}=(P_{\text{Length}}\times T_{TX}\times I_{TX}\times V_{TX})\times (P_{TX_{i}}+P_{RTX_{i}})$$

where *P_TXi_* is the total number of packets transmitted by node *i*. *P_RTXi_* is the total number of retransmitted packets by node *i*, due to the ACK packet.

Moreover, *E_RXi_*. will be the receiving energy in node *i*, then,

(14) $$E_{RX_{i}}=(P_{\text{Length}}\times T_{RX}\times I_{RX}\times V_{RX})\times P_{RX_{i}}$$

where *P_RXi_* is the total number of packets received by node *i*.

In the same way, *E_Switchingi_*, the switching energy, will be spent when a node switches between transmission and receiving or *vice versa.* The *E_Switchingi_* in node *i* is given by,

(15) $$E_{\text{Switchin}g_{i}}=(T_{\text{Switching}}\times I_{\text{Switching}}\times V_{\text{Switching}})\times (P_{TX_{i}}+P_{RTX_{i}}+P_{RX_{i}})$$

The term (*P_TXi_* + *P_RTXi_* + *P_RXi_*) represents the number of times there is a change of activity (switching) from sending to receiving a packet or *vice versa*. Therefore, when a node sends a packet, in the best case, an acknowledgment is received, then *P_TXi_* = 1 and *P_RXi_* = 1. The node received the ACK, so it did not send a retransmission of the packet, then *P_RTXi_* = 0. It is necessary to note that this switching operation is done in two separate steps: the node uses switching energy to change the state, transmits the packet, and again, it uses switching energy and finally receives the packet. The transmission and receiving energies are considered by Equations (13) and (14), respectively.

For each transmitted packet, node *i* runs the CSMA/CA algorithm. The corresponding consumed energy, *E_CSMAi_*, is given by,

(16) $$E_{\text{CSM}A_{i}}=(T_{\text{CSMA}}\times I_{\text{CSMA}}\times V_{\text{CSMA}})\times (P_{TX_{i}}+P_{RTX_{i}}+N_{RT_{i}})$$

where *N_RTi_* is the number of times that the CSMA/CA algorithm backs off since the first time the channel was found busy. It is a random variable, that is it takes a different value in each transmission. The term (*P_TXi_* + *P_RTXi_* + *N_RTi_*) implies that before each packet is transmitted, it must go through before the channel listening process (CSMA/CA algorithm). In the best case, the term (*P_TXi_* + *P_RTXi_* + *N_RTi_*) will have *P_TXi_* = 1, with the channel free from the first time, then *N_RTi_*. = 0 (there were no retries listening to the channel), and the packet is successfully transmitted, then *P_RTXi_* = 0 (no retransmissions). However, network conditions are not always the best, and there will be collisions and, hence, packet retransmissions and retries in listening to the channel, then the *N_RTi_* and *P_RTXi_* variables will take non-zero values.

Throughout the sampling time (*T_Sampling_*), the node consumes microcontroller energy in an active mode for this model. Therefore,

(17) $$E_{MC_{i}}=T_{\text{Sampling}}\times I_{MC}\times V_{MC}$$

Therefore, when the sampling time starts, all of the nodes start, so that the starting energy consumed by node *i* is *E_Startingi_ = T_Starting_ × I_Starting_ × V_Starting_*. Finally, when the time is over, node *i* turns off, consuming shutdown energy, so *E_Shutdowni_* = (*T_Shutdown_ × I_Shutdown_ × V_Shutdown_*).

In this way, at the end of the sampling time, the node has a total cumulative energy consumed by all of the functions that were performed during the network processes. Therefore,

(18) $$\text{nodeEnerg}y_{i}=E_{\text{Startin}g_{i}}+E_{MC_{i}}+E_{\text{Shutdow}n_{i}}+E_{\text{Switchin}g_{i}}+E_{\text{CSM}A_{i}}+E_{TX_{i}}+E_{RX_{i}}$$

If we want to obtain the total network energy, we add the total energy of each node when the sampling time is over, as follows:

(19) $$\text{totalEnergy}=\overset{\text{totalNodes}}{\underset{i=1}{\text{∑}}}\text{nodeEnerg}y_{i}$$

where *totalNodes* is the total number of nodes.

**Figure 16.**
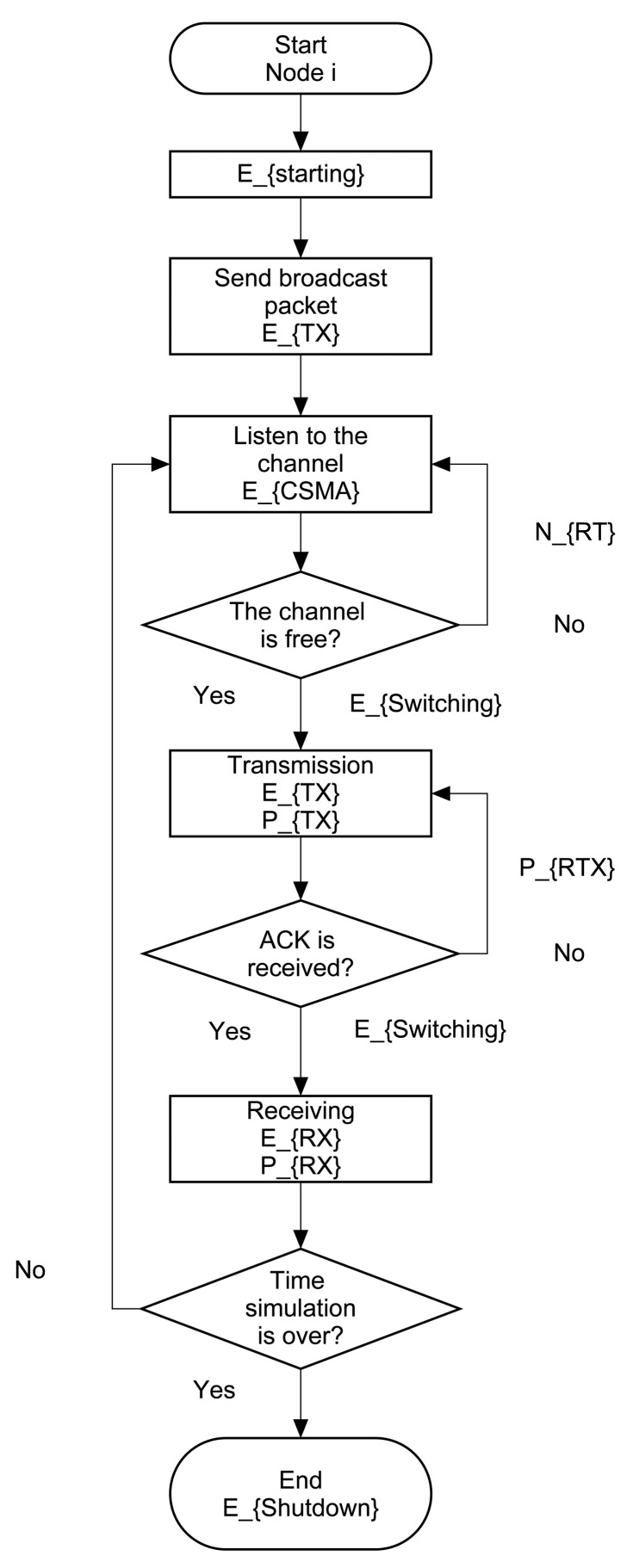
Flowchart scheme of the energy model for node *i*.

The flowchart in Figure 16 describes the actions taken by node *i* since it switches on (consuming starting energy, *E_Startingi_*) and is part of a network, to the time it shuts down. The most complex stage is when the node listens to the channel. This activity causes the node to transmit or not. All sent packets (transmitted packets or retransmissions) imply that it must first listen to the channel, *i.e*., the node ran the CSMA/CA algorithm using CSMA energy (*E_CSMAi_*). If the channel is free, the node uses transmission energy (*E_TXi_*) and transmits the packet (*P_TXi_*). If the channel is busy, the node generates a retry variable (*N_RTi_*), because it has to run the CSMA/CA algorithm again. After the node sends the packet, if it receives an ACK, the receiving energy is used (*E_RXi_*) and receives the packet (*P_RXi_*). In contrast, if the node does not receive an ACK, it retransmits the generated packet as a retransmission (*P_RTXi_*). In all changes of activity, the node consumes switching energy (*E_Switchingi_*). After this basic cycle of a single packet, the node continues receiving, listening, transmitting and switching states, until the simulation time is over and the node turns off using shutdown energy (*E_Shutdowni_*). During all performed processes by a node as part of a network, it is in active node, and the microcontroller stays on throughout the sampling period, consuming microcontroller energy (*E_MCi_*).

##### Energy

**Figure 17.**
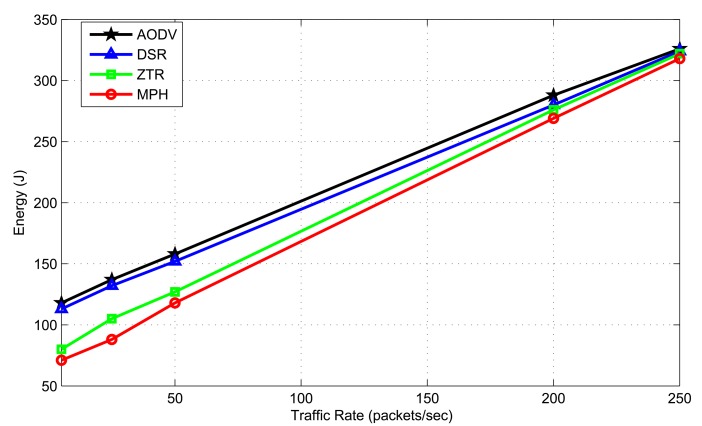
Global energy consumption.

As we have pointed out, a very important metric to measure network performance is the energy A low energy consumption allows reduced maintenance costs in the network; the sensors are able to supply much information, and then, the network will be scalable and long-lived across time. Figure 17 shows the total energy consumption for the topology shown in Figure 10 for each of the mentioned protocols and ZTR. The authors established the range of a node of 8 m and simulated the energy consumption for each of the protocols under different traffic rates.

We see that for traffic rates, such as 5, 25 and 50 packets/s, MPH and ZTR have a very good energy performance with respect to AODV and DSR. However, when the traffic becomes higher, such as 250 packets/s, the energy consumption of MPH and ZTR is closer to AODV and DSR consumption, possibly because of collisions, retransmissions and forwarding of packets that exceed the network capacity. Because of the hierarchical routing, ZTR and MPH have similar energy consumption, showing that the MPH protocol competes with other multi-hop network protocols in sensor networks, and it has an energy performance similar to that one of a simple routing tree. We can calculate that MPH protocol shows a reduction of 15.9% on energy consumption with respect to AODV, 13.7% with respect to DSR and 5% compared with ZTR.

The MPH protocol takes the minimum number of hops to reach the coordinator (destination). This may occur, because sometimes, the AODV and DSR protocols do not have the shortest path to the destination, because this route has not been updated or because the shortest route has expired and a node took a back-up route. ZTR does not always guarantee the route with fewer hops, and if a node is disconnected temporarily, all of its child nodes are cut off in the upward path, resulting in an increased number of retransmissions in the MAC layer. MPH always guarantees the shortest path, and thus, it produces smaller delays and fewer packet delivery errors, resulting in less energy consumption.

**Figure 18.**
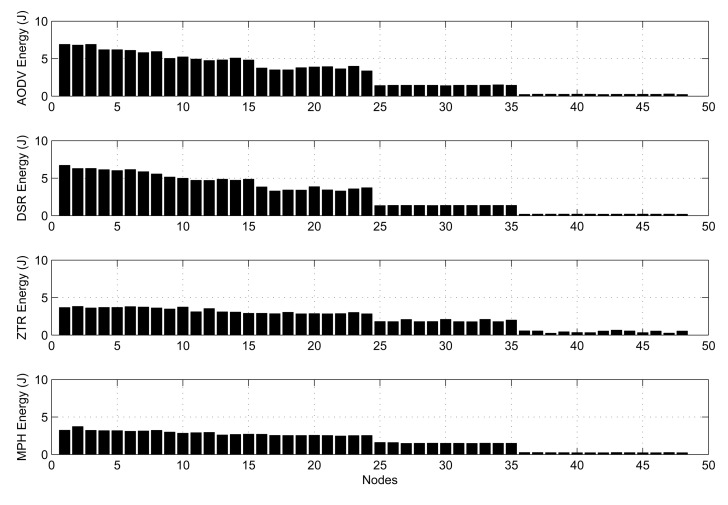
Local energy consumption.

The local energy consumption is presented in Figure 18. Local energy is an important metric to analyze how energy is distributed over the network. In Figure 18, we show the total energy in each node for the different protocols. For AODV and DSR, we see that the energy consumption is similar for all of the nodes in the same ring around the coordinator. As expected, the rings closer to the sink spend more energy, since they carry their own packets and transport packets from farther nodes. ZTR has some nodes with higher energy consumption than the other nodes in the same ring; this is because, if a node is temporarily disconnected, the child nodes have no route to send packets to the coordinator and generate more transmission retry packets. In the MPH protocol, the energy of nodes is distributed more uniformly, but still consuming the greatest energy in nodes belonging to rings near the sink node.

**Figure 19.**
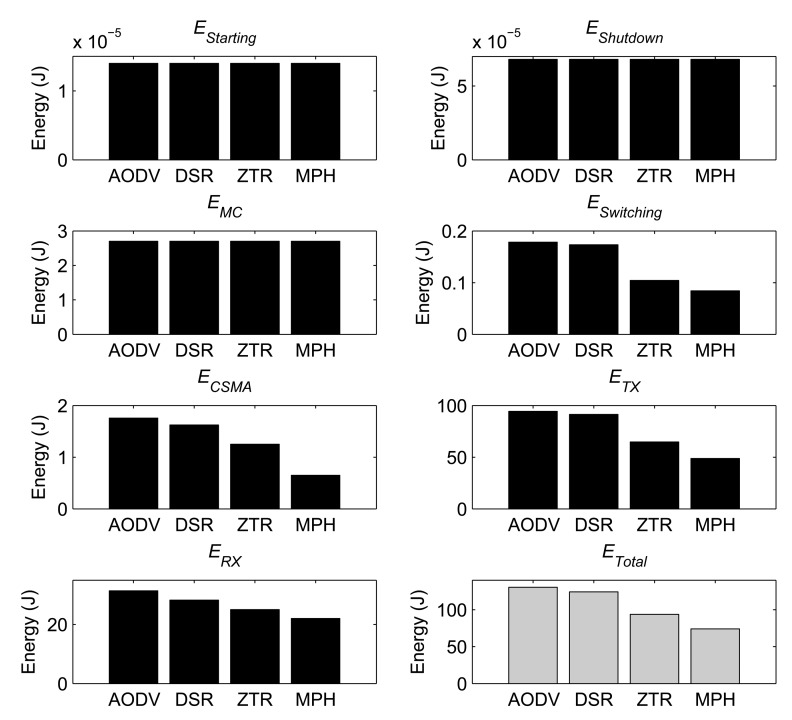
Consumed energies for AODV, DSR, ZTRand MPH.

In Figure 19, the consumed energies explained through Equations (4) to (10) are shown. Here, we implemented the energy model for the four studied protocols: AODV, DSR, ZTR and MPH. The packet rate contributed for each node is 20 packets/s. We see the cost of each protocol for each of the types of tested energies. Thus, we prove that the transmitting and receiving energies are the most influential in the total energy, and this situation becomes critical, because they provide the communication among nodes.

## 5. Performance Comparison of the WSN with Different Spatial Node Distributions

We compared three different scenarios in order to observe the impact on system performance when we change the topology arrangement. Let us consider using a transmission power of 0 dBm [34] for the active transmission mode according to the CC2530 chip [32] and a receiving power of −85 dBm according to the IEEE standard 802.15.4 [33]. With these power values, we obtain an average coverage radio of 50 m for sensors following Equation (20) for outdoors [33].

(20) $$d=8*10^{\frac{P_{t}-P_{r}-58.5}{33}}$$

where *d* is the coverage radio, *P_t_* is the transmission power in dB and *P_r_* is the receiving power in dB.

Table 4 shows the parameters in order to simulate the three scenarios. Below are three tables, one for each of the proposed scenarios. For each scenario, the studied metrics are: retransmissions, CSMA retries, overhead, discovered routes, recovery time and energy. We should note that the tables contain average values taken over a sampling period of 100 s in the same conditions as in the Section 4. The recovery time metric was analyzed with respect to the worst case, *i.e*., when 50% of the network nodes are turned off. Retransmissions and CSMA retry metrics show average values. Overhead and the discovered routes metrics are shown in percentage. Finally, energy is described in Joules.

Tables 5, 6 and 7 show the results of average values for the studied metrics in this work. We can observe that the scenario that negatively affects the three protocols is the random uniform deployment. This is because the number of neighbors for each node may vary greatly with respect to the other two configurations. In the former topology, nodes more distant from the coordinator may have longer routes, and the random distribution of nodes may cause the appearance of areas of high node density, causing more collisions in those nodes.

### 5.1. A Grid Where the Coordinator is Located in One Corner

Table 5 shows the results for a grid of 49 nodes where the coordinator is located in a corner of the grid and all nodes generate and send traffic to it. The coverage radio is 50 m and the area is 150 m × 150 m. Results show that the MPH protocol has a better performance in all metrics with respect to the other three protocols. Regarding the retransmission metric, MPH is 19% less than AODV, 17% less than DSR and 18% less than ZTR. With regard to the CSMA retry metric, MPH shows an average reduction of 30% against AODV, 27% against DSR and 29% against ZTR. Concerning the overhead metric, MPH is 27% less than AODV and 17% compared to DSR. MPH has only 11% more overhead with respect to ZTR. About the discovered routes metric, MPH has a greater average discovered routes, such as 43% against AODV, 28% against DSR and 6% with respect to ZTR. Regarding the recovery time metric, MPH is faster in the recovery of the topology, having 41% over AODV, 39% over DSR and 35% relative to ZTR. Finally, with regard to energy, MPH has an energy savings of 16% compared with AODV, 14% against DSR and 5% in relation to ZTR.

### 5.2. Non-Uniform Random Distribution

Table 6 exposes the results for a grid of 49 nodes where the coordinator is located in the center of the topology and all nodes generate and send traffic to it. The coverage radio is 50 m and the area is 150 m × 150 m. This scenario shows the greater amount of traffic in the center, because the coordinator is the destination. Moreover, we randomly put 40% of nodes near the central part of the topology, and the other nodes are distributed along the whole area in a random way. Results show that the MPH protocol has better performance in all metrics with respect to the other three protocols again. Regarding the retransmission metric, MPH is 20% less than AODV, 19% less than DSR and 19% less than ZTR. With regard to the CSMA retry metric, MPH exhibits an average reduction of 22% against AODV, 18% against DSR and 25% against ZTR. Concerning the overhead metric, MPH is 28% less than AODV and 23% compared to DSR. MPH has only 12% more overhead with respect to ZTR. Regarding the discovered routes metric, MPH has a greater average discovered routes, such as 37% more than AODV, 26% more than DSR and 5% more than ZTR. Regarding the recovery time metric, MPH is faster in the recovery of the topology, having 40% over AODV and DSR and 33% relative to ZTR. Finally, with regard to energy, MPH has an energy savings of 21% compared with AODV, 18% against DSR and 12% in relation to ZTR. Here, we note that MPH is more efficient in this type of scenario with respect to the energy consumption, because routes are better distributed along nodes.

### 5.3. Uniform Random Distribution

Table 7 reveals the results for 49 nodes under random positions along a 150 m × 150 m area. All nodes generate and send traffic to the coordinator. The coverage radio is 50 m. The results confirm that this is the worst case among the three presented scenarios. Nevertheless, the MPH protocol has a better performance in all metrics with respect to the other three protocols. Regarding the retransmission metric, MPH is 24% less than AODV, 18% less than DSR and 19% less than ZTR. With regard to the CSMA retry metric, MPH shows an average reduction of 27% against AODV and DSR and 25% against ZTR. Concerning the overhead metric, MPH exhibits 33% less than AODV, 31% less that DSR, and now, MPH is better, at 4% with respect to ZTR. About the discovered routes metric, MPH has a greater average discovered routes, such as 48% more than AODV, 40% more than DSR and 5% more than ZTR. Regarding the recovery time metric, MPH is faster in the recovery of the topology, being 27% over AODV, 26% over DSR and 9% relative to ZTR. Finally, with regard to energy, MPH has an energy savings of 16% compared with AODV, 14% against DSR and 5% in relation to ZTR, showing a similar behavior as in the first scenario.

## 6. Conclusions

In this work, the authors presented various performance metrics for wireless sensor networks using AODV, DSR, ZTR and the proposed MPH routing protocol. Besides, we compared the energy efficiency of MPH with that of AODV, DSR and ZTR. We tested our MPH protocol in a simulator using conditions taken from a real environment and evaluated its performance for 10 days for 10 specific nodes chosen randomly. The simulation results were compared with the corresponding measurements gathered from the real network. This comparison showed that the simulator was well programmed and gave results very close to those of the real network under the conditions and parameters used. This proved that it was a reliable tool to simulate different conditions of a WSN.

We then analyze metrics that have a direct impact on the energy consumption of a network. Retransmissions, retries to access the channel, the number of discovered routes in the tables of the nodes, the recovery time after a failure in some links of a topology that already has valid routes: all these are parameters that affect the local and global energy consumption. The MPH protocol was designed for a scenario where there is a single, robust, high processing node, called the coordinator node, and the other nodes have low memory and low processing capabilities. The aforementioned metrics were calculated and analyzed for this particular scenario, and the performances of AODV, DSR, ZTR and MPH were evaluated under the same conditions of topology and network design, using an energy model proposed by us. The MPH protocol showed great advantages over the other protocols regarding network reconfiguration under adverse conditions. This was so because MPH has a reactivity mechanism to cope with changes in the network and enjoys a smooth route maintenance mechanism that enters into action when the links are connected intermittently. In addition, the developed energy model presents better performance under the MPH protocol, because this protocol always guarantees the shortest route to the destination, which translates into lower packet delays and fewer packet retransmissions, because there are fewer intermediate nodes and also because MPH has more valid routes, so that packets are forwarded correctly most of the time.

In this paper, we also compared the performance of MPH with that of a simple routing algorithm, such as ZTR, in order to show that MPH is a highly competitive protocol in WSNs, with good performance as measured by the results in the metrics considered and with an energy consumption equivalent to that of a less complex routing algorithm, such as ZTR. Besides, we could show that even though MPH presents more elaborate neighbor tables, this does not represents higher complexity compared with a simple solution in WSNs, such as ZTR.

## Figures and Tables

**Table 1.** Simulation and real network parameters. CSMA/CA, carrier sense multiple access with collision avoidance.

**Parameter**		**Value**
**Physical Layer Parameters**		

Sensitivity thresholds		−78 to −94 dBm
Transmission power		4.5 dBm

**MAC Layer Parameters**		

Waiting time for NDRACKpacket		30 ms
Maximum retransmission number		3
Maximum retry number		5
Maximum number of tries to reach a node from the coordinator		9
Packet error rate		1%
Average frame length		22 bytes
Maximum number of backoffs		4
MAC protocol		IEEE 802.15.4
MAC layer		CSMA/CA

**Network Layer Parameters**		

Number of nodes		99
Maximum number of neighbors		16
Discovery neighbor time		30 s
Update time neighbors table		30 ms
Maximum data rate		250 kbps
Routing		Hierarchical
Scenario		Static nodes

**Table 2.** Parameters for the tested scenarios.

**Parameter**	**Value**
Maximum data rate	250 kbps
Discovery packet time	10 s
Receiver sensitivity	−85 dBm
Average frame length	22 Bytes
MAC layer	CSMA/CA
Maximum number of backoffs	4
Packet loss	1%
Scenario	Static

**Table 3.** Energy model [34].

	**Voltage (mV)**	**Current (mA)**	**Time (ms)**	**Energy (J)**
Start-up mode	120	12	0.2	*E_Starting_* = 0.000288
MCU running on 32-MHz clock	75	7.5	1.7	*E_MC_* = 0.000956
CSMA/CA algorithm	270	27	1.068	*E_CSMA_* = 0.00778
Switch from RX to TX	140	14	0.2	*E_Switching_* = 0.000392
Switch from TX to RX	250	25	0.2	*E_Switching_* = 0.00125
Radio in RX mode (processing and waiting)	250	25	4.1915	*E_RX_* = 0.0262
Radio in TX mode	320	23	0.58	*E_TX_* = 0.00426
Shut down mode	75	7.5	2.5	*E_Shutdown_* = 0.00141

**Table 4.** Parameters for the tested scenarios.

**Parameter**	**Value**
Maximum data rate	250 kbps
Discovery packet time	10 s
Receiver sensitivity	−85 dBm
Average frame length	22 Bytes
MAC layer	CSMA/CA
Maximum number of backoffs	4
Packet loss	1%
Transmission power	0 dBm
Coverage radio	50 m
Scenario	Static

**Table 5.** Grid topology where the coordinator is located in one corner.

**Topology of Figure 10 Where the Coordinator is Located in One Corner**						

	**Retransmissions**	**CSMA Retries**	**Overhead (%)**	**Discovered Routes (%)**	**Recovery Time (s)**	**Energy (J)**
AODV	2.12	2.44	43.4	51.7	34	205.4
DSR	2.07	2.34	38.2	67.1	33	200.2
ZTR	2.08	2.39	28.2	89.0	31	182.0
MPH	1.71	1.70	31.7	94.8	20	172.8

**Table 6.** Non-uniform random distribution.

**Non-Uniform Random Distribution**						

	**Retransmissions**	**CSMA Retries**	**Overhead (%)**	**Discovered Routes (%)**	**Recovery Time (s)**	**Energy (J)**
**AODV**	1.92	2.09	40.1	60.3	30	192.3
**DSR**	1.90	1.98	37.3	70.1	30	184.3
**ZTR**	1.88	2.15	25.3	91.0	27	172.7
**MPH**	1.53	1.62	28.7	95.5	18	151.4

**Table 7.** Uniform Random Distribution.

**Uniform random distribution**						

	**Retransmissions**	**CSMA Retries**	**Overhead (%)**	**Discovered Routes (%)**	**Recovery Time (s)**	**Energy (J)**
**AODV**	2.34	2.61	50.5	48.3	40	221.7
**DSR**	2.17	2.59	48.7	55.1	39	216.1
**ZTR**	2.20	2.55	35.2	87.3	32	197.3
**MPH**	1.78	1.90	33.6	92.2	29	186.5
